# Transferrin protects against Parkinsonian neurotoxicity and is deficient in Parkinson’s substantia nigra

**DOI:** 10.1038/sigtrans.2016.15

**Published:** 2016-09-02

**Authors:** Scott Ayton, Peng Lei, Catriona Mclean, Ashley I Bush, David I Finkelstein

**Affiliations:** 1 The Florey Institute for Neuroscience and Mental Health, The University of Melbourne, Parkville, Victoria, Australia; 2 Department of Neurology, State Key Laboratory of Biotherapy, West China Hospital, Sichuan University, and Collaborative Innovation Center for Biotherapy, Chengdu, Sichuan, China; 3 The Alfred Hospital, Department of Anatomical Pathology, Melbourne, Victoria, Australia

## Abstract

Iron deposition in Parkinson’s disease (PD) is a potential disease-modifying target. We previously showed that supplementation of the iron-exporter, ceruloplasmin, selectively corrected nigral iron elevation in the 1-methyl-4-phenyl-1,2,3,6-tetrahydropyridine (MPTP) model. Ceruloplasmin delivers iron to transferrin (Tf), the extracellular iron-transporting protein. We show that Tf protein levels are decreased in the nigra of post-mortem PD brains compared with controls (−35%; *n*=10 each). Because Tf traffics iron away from iron-replete tissues, we hypothesized that Tf supplementation could selectively facilitate iron export from the nigra in PD. In cultured neurons, Tf treatment corrected iron accumulation, and subcutaneous Tf to mice ameliorated iron accumulation and motor deficits in the MPTP model of PD. Although these data support a role for Tf in the disease mechanism for PD, and its potential use for correcting disorders of iron overload, Tf therapy also caused systemic iron depletion, which could limit its application for PD.

## Introduction

Iron deposition in the Parkinson’s disease (PD) substantia nigra^1^ (SN) could be a disease-modifying target. A recent phase II trial of the iron chelator, deferiprone, reported improved motor outcomes associated with lowering of nigral iron in PD patients.^2^ Deferiprone holds great promise for PD as a disease-modifying therapy; however, iron-chelating compounds, developed for disorders of peripheral iron accumulation, could remove iron from tissues unaffected by the disease or have other side effects. Indeed, some iron-chelating compounds have been shown to be nephrotoxic,^3^ cause neutorpeinia and agranulocytosis,^4^ reduce immunological function,^5^ and reduce striatal dopamine.^6^ Deferasirox, a major iron chelator drug, has the second highest rate of deaths on treatment for any drug approved by the Food and Drug Administration.^7^ Although lower doses of deferiprone used in clinical trials of neurodegenerative diseases have shown favorable safety profiles, there is an opportunity to develop drugs that more selective target pathological iron accumulation in PD, which could limit side effects that might develop with long-term treatment for this illness.

We recently showed that the iron-exporting protein, ceruloplasmin, is dysfunctional in PD-affected nigra, and ceruloplasmin supplementation selectively restored nigral iron accumulation in the 1-methyl-4-phenyl-1,2,3,6-tetrahydropyridine (MPTP) model of PD.^8,9^ Ceruloplasmin receives iron from the iron-exporting protein, ferroportin; the expression of ferroportin on the neuronal membrane in PD is limited by the reduced abundance of tau and the amyloid precursor protein, which combine to stabilize the surface presentation of ferroportin.^10,11^ Ceruloplasmin oxidizes ferrous iron from ferroportin and delivers ferric iron to the extracellular iron transporter, transferrin (Tf), for redistribution throughout the body. Iron levels are maintained in the cell by balancing cellular uptake with efflux, where Tf is required for both mechanisms.^12,13^ Tf binds to transferrin receptors when the cell requires iron and removes iron from the ferroportin/ceruloplasmin complex when the cell is iron replete.

We reasoned that, like ceruloplasmin,^8^ supplementation of Tf could also be used as a drug to correct iron elevation in PD with superior selectivity compared with small-molecule chelators. Peripheral Tf also has regulated access to the brain by receptor-mediated transcytosis.^14–16^ We sought to exploit these properties of Tf and determine whether supplemental Tf administration is safe and effective in redistributing iron out of cultured cells and in mice, and its possible use as a therapeutic for PD.

## Materials and methods

### Ethics statement

For studies involving human tissue, the collection, processing and storage of human brains for research purposes were conducted by the Victorian Brain Bank Network. Ethics approval was provided by The University of Melbourne Human Research Ethics Committee, application number 941478X, titled ‘Brain Banking for Neuroscience Research’. Donors during life or their notarized next of kin gave written consent for the removal of their brain and up to 20 ml of cerebrospinal fluid from the brain. The written consent is for the use of tissues for the purpose of medical research.

All animal experiments were approved by the Howard Florey Animal Ethics Committee and were conducted in accordance with state law and the Australian Code of Practice for the Care and Use of Animals for Scientific Purposes as described by the National Health and Medical Research Council of Australia.

### Post-mortem SN tissue

The collection, processing and storage of human brains for research purposes were conducted by the Victorian Brain Bank Network. Post-mortem brain samples were from 10 control individuals (5 male and 5 female; 76.6±2.1 years), with no known history of neurological or psychiatric disorders and minimal neuronal pathology, and 10 patients with clinically defined PD (6 male and 4 female; 75±2.0 years), confirmed by the loss of nigral neurons and the presence of Lewy bodies.^8^

Dissected brain tissue was homogenized in phosphate-buffered saline with protease inhibitors (Pierce (TM) protease inhibitors, Thermo Fisher Scientific, MA, USA) and phosphatase inhibitors (Sigma, St Louis, MO, USA). Iron was measured in homogenate by atomic absorption spectrometry (AAS) as previously described^8^ and Tf was measured by western blot.

### Cell culture

BE(2)-M17 human neuroblastoma cells (M17 cells) were cultured in Optimem (Gibco/Thermo Fisher Scientific, MA, USA) media. The media was supplemented with 10% fetal bovine serum, 10 000 U ml^−1^ penicillin G sodium (Gibco), 10 000 μg ml^−1^ streptomycin sulfate (Gibco) and 25 μg ml^−1^ amphotericin B (Gibco). Cells were grown in T75 cm^2^ flasks (Nunc/Thermo Fisher Scientific, MA, USA) maintained at 37 °C and 5% carbon dioxide.

#### Tf(^59^Fe)_2_ preparation

Human apo-Tf (Sigma) was treated with sodium ascorbate to remove all trace of unlabeled Fe. Apo-Tf was then loaded with ^59^Fe (PerkinElmer, Boston, MA, USA).

#### Tf(^59^Fe)_2_ release

M17 cells were plated onto 12-well plates and incubated for 24 h with 1.0×10^−6^ M Tf(^59^Fe)_2_ in Optimem media (Invitrogen/Thermo Fisher Scientific, MA, USA) without serum. Media was then removed and cells were washed twice in Hanks' buffered saline solution (HBSS) (Invitrogen). Cells were then incubated in HBSS alone±1 μM or 10 μM apo-Tf. Media was collected after 3 h and measured for ^59^Fe release by γ-counter (Wizard 3, PerkinElmer) and expressed as counts per min. Cells were washed with HBSS, detached with trypsin and ^59^Fe measured by γ-counter.

#### Apo-Tf treatment in MPP+ intoxicated cells

The MTT (thiazolyl blue tetrazolium bromide) assay was used to measure cell viability in response to MPP+ (toxic metabolite of MPTP) intoxiciation and Apo-Tf treatment. M17 cells were plated onto 96-well plates; after culturing for 24 h, serum-containing (10%) Optimem media (Invitrogen) was replaced with serum-free media. Cells were treated with MPP+ (Sigma) 0–2.5 mM for 24 h in the absence and presence of 20 μM Apo-Tf (Sigma). At the 21-h time point, 98% MTT (Sigma) was added to cell media (300 μg ml^−1^). After 24 h exposure to MPP+, the media was removed and replaced with 100 μl dimethyl sulfoxide, before measuring the absorbance at 540 nm of the samples.

### Mice

All mice were housed according to the standard animal care protocols and fed standard laboratory chow and tap water *ad libitum*. C57Bl6 mice were obtained from Monash animal services.

#### MPTP

Five-month-old C57/Bl6 male mice were obtained from Monash animal services and administered Apo-Tf (Sigma) and/or MPTP. MPTP was administered by intraperitonealIP in 4×12.5 mg kg^−1^ doses, 2 h apart. Apo-Tf dissolved in saline was administered subcutaneously at a dose of 30 mg kg^−1^ every 3 days, beginning the day after MPTP administration. Mice were assessed on the pole test on days 19 and 20, and were killed 21 days after MPTP administration (sodium pentobarbitone 100 mg kg^−1^) and perfused with phosphate-buffered saline. The SN was removed for iron analysis (AAS).

#### Chronic Tf treatment

Mice were injected subcutaneously with 30 mg kg^−1^ Apo-Tf (Sigma) in saline every third day for 3 weeks. After 21 days, mice were culled and perfused, and their brains and other organs were dissected and measured for iron (AAS). Blood was collected in lithium-heparin tubes. Hematological characterization of blood was performed by Gribbles Veterinary Pathology (Gribbles Veterinary Australia, accreditation to ISO/IEC 17025 conferred by the National Association of Testing Authorities and certification to AS/NZS 9001: 2008 conferred by Lloyd’s Register Quality Assurance).

#### Pole test

Mice were placed with heads facing upwards at the top of a vertical rough surfaced pole (dimensions: 30 cm length and 1 cm diameter), with the base plate of the pole placed in their home cage. Mice were tested for 2 days, habituation day (day 1) and test day (day 2) allowing five consecutive trials on the pole. The mice were assessed on their ability to completely turn 180° towards their home cage (time to turn) and the time taken to reach the base of the pole (time to complete). Animals were recorded via digital video on the test day with a stopwatch in view. The videos were analyzed using slow motion playback (Windows Media Player Classic). Measurement of the turn began immediately before the commencement of the turn. The quickest of the five trials for each was used for analysis.

### Iron quantification

Aliquots of homogenized human or mouse tissue, or human cerebrospinal fluid were lyophilized before the addition of 30 μl 69% nitric acid (Aristar-ultraclean grade) and incubated at 90 °C for 20 min. A volume of 30 μl of hydrogen peroxide (30%, Merck, Kenilworth, NJ, USA) was added to the sample before further treatment at 70 °C for 20 min. The samples were diluted in double-distilled water and loaded on a Varian AA240 AAS (Palo Alto, CA, USA).

### Western blot protein quantification

Sample homogenate was separated by 4–12% bis-tris gels (Invitrogen) and electroblotted on a nitrocellulose membrane (iBlot; Invitrogen). Tf antibody (1:1000 dilution; Alpha Diagnostic International (San Antonio, TX, USA), catalog number: TF11-A) was imaged by enhanced chemiluminescence (GE Healthcare, Little Chalfont, UK) and scanned using Fujifilm LAS-3000 (Tokyo, Japan). Membranes of tissue samples were re-probed with β-actin antibody (1:10 000 dilution; Sigma catalog number: A5441).

### Statistics

All statistical procedures were performed with SPSS version 14.0 software (Lead Technologies, Charlotte, NC, USA). *T*-tests (two tailed) were performed in each instance unless stated.

## Results

### Tf is reduced in PD

**Figure 1:**
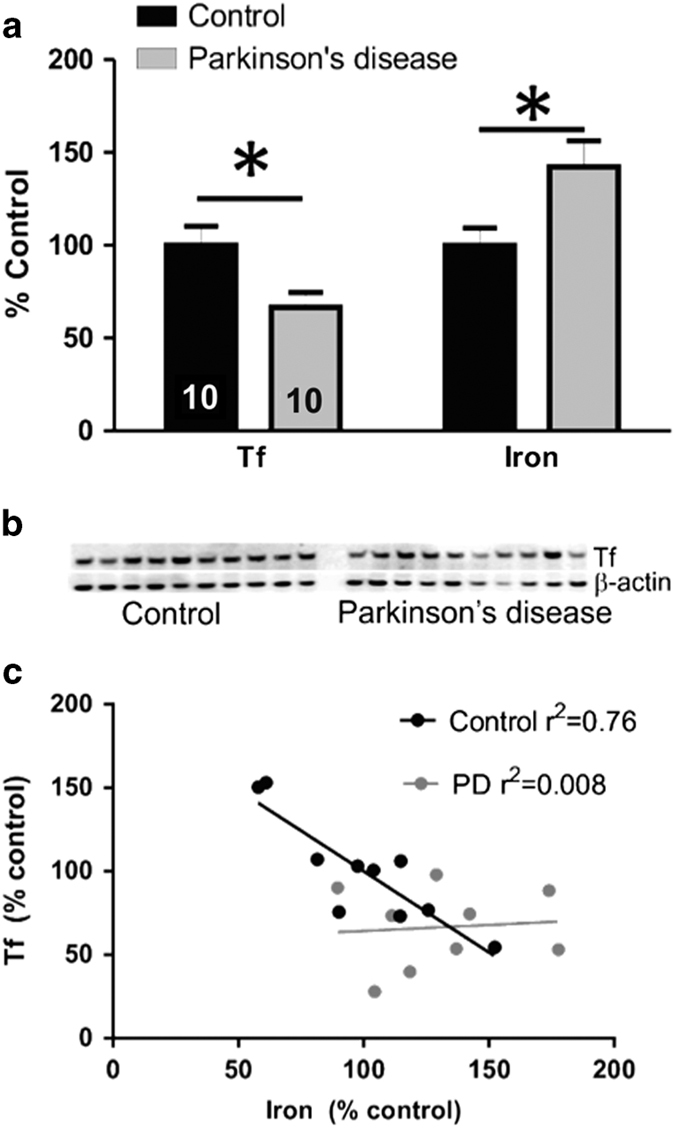
Tf is reduced in PD substantia nigra. (**a**) Iron (AAS) and Tf (western blot) levels in nigral of control and PD patients at autopsy. Representative western blot shown in **b**. (**c**) Scatter plots of iron and Tf in SN of PD cases and controls (100%=mean of control values). Tf was inversely correlated with iron in controls (Pearson’s regression: *P*=0.001, *r*^2^=0.76), but not PD cases (Pearson’s regression: *P*=0.8, *r*^2^=0.008). Data are means±s.e.m. *P<0.05.

A decrease in serum Tf has previously been reported in PD,^17^ whereas a prior paper observed no difference in Tf in PD or AD SN, which also reported no change in iron compared with elderly controls.^18^ We examined SN from post-mortem PD subjects (*n*=10) and controls (*n*=10), and found the loss of Tf in disease (−35%, *P*=0.021; Figures 1a and b), accompanying iron elevation (+42%, *P*=0.021; Figure 1a) as previously described.^8,10^ The loss of Tf could restrict iron flux in this nucleus, and contribute to iron deposition. We examined this further using regression analysis. In controls, Tf was inversely correlated with iron levels (Pearson’s correlation*; r*^2^=0.76, *P*=0.001; Figure 1c). In PD cases, however, the relationship between Tf and iron was lost (Pearson’s correlation*; r*^2^=0.008, *P*>0.05). As Tf facilitates iron flux, the inverse relationship observed in controls is consistent with Tf expression facilitating iron efflux.

### Tf helps maintain iron balance in cultured neuronal cells

**Figure 2:**
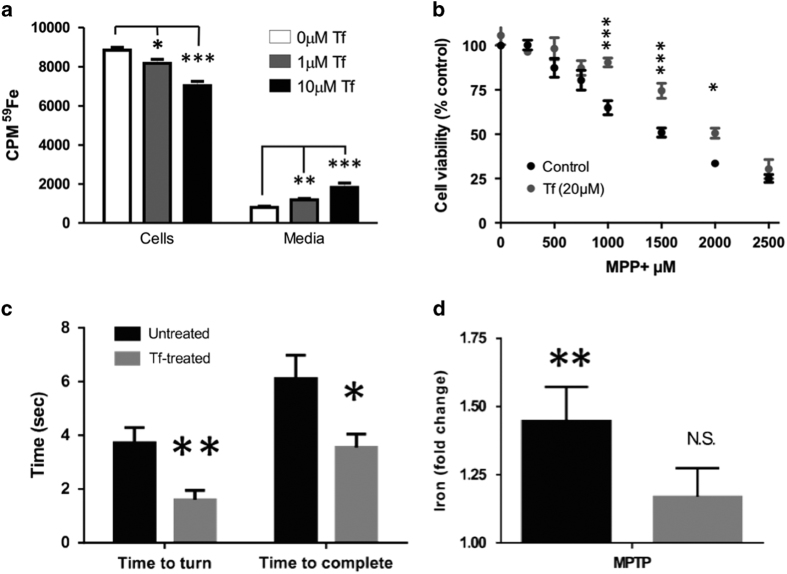
Tf supplementation simulates iron release from iron-replete cells and protects against MPTP in cells and mice. (**a**) Iron release from ^59^Fe-loaded M17 cells was measured by γ-counter. (**b**) M17 cells were treated with MPP+ in the absence and presence of apo-Tf for 24 h. Cell viability assessed using the MTT assay. (**c**, **d**) MPTP (50 mg kg^−1^) was administered to mice. A subgroup of mice was subsequently administered TF (30 mg kg^−1^; intraperitoneal) every third day for 21 days and compared with controls (*N*=9 each). (**c**) Mice performed the pole test 20 days after MPTP lesion. (**d**) Iron (AAS) content in nigra of MPTP and Tf-treated mice expressed as fold elevation of controls. Data are means±s.e.m. *P<0.05, **P<0.01.

To demonstrate that Tf could promote iron export of neurons, we loaded dopaminergic M17 cells with ^59^Fe then washed the cells and replaced the media (HBSS-no serum) with 0, 1 or 10 μM apo-Tf (*n*=8 each). After 3 h of treatment, ^59^Fe was measured in cells and in the media. The ^59^Fe content in cells was reduced in cells with 1 μM Tf (*P*=0.042) and 10 μM Tf (*P*<0.001) compared with control-treated cells (analysis of variance; Figure 2a). Reciprocally, ^59^Fe content in the media was elevated above controls with 1 (*P*=0.006) and 10 μM (*P*=0.001; analysis of variance; Figure 2a), demonstrating that apo-Tf, like synthetic iron chelators, is effective in removing iron from cells.

### Tf protects against MPP+ intoxication

The MPTP model of PD recapitulates selective iron accumulation in the SN and can be rescued by iron removal.^8,19^ We tested whether stimulating cellular iron export could protect cultured neurons against MPP+ (toxic metabolite of MPTP). We observed that Apo-Tf treatment (20 μM) protected against MPP+-induced toxicity of M17 cells (two-factor analysis of variance, *P*<0.001; Figure 2b). We chose to use a slightly higher dose of apo-Tf in the MPP+ paradigm (20 μM) compared with that of the non-intoxicated cells presented in Figure 2a (1–10 μM) because Tf-mediated iron efflux is impaired in PD, and in PD models.^8,10,11^

### Tf supplementation reverses MPTP-induced disability and iron elevation

We explored whether apo-Tf supplementation could rescue iron accumulation and behavioral disability in the MPTP mouse brain model, as peripheral Tf enters the brain by receptor-mediated transcytosis.^14–16^ MPTP (50 mg kg^−1^; intraperitoneal) was administered to mice, and a subgroup was subsequently administered Tf (30 mg kg^−1^ every 3 days subcutaneously; *n*=9). MPTP causes disability on the pole test,^20^ as measured by increased time to turn 180° (time to turn), and increased time to return to the home cage (time to complete). We found that apo-Tf treatment improved time to turn (*P*=0.005) and time to compete (*P*=0.022; Figure 2c). Improvement in motor performance was associated with normalization of iron elevation in SN (Figure 2d). MPTP caused nigral iron accumulation (~1.45-fold, *P*<0.001) in non-TF-treated mice; however, iron elevation was attenuated in apo-Tf-treated mice, which did not have significant elevation in nigral iron, following MPTP compared with non-MPTP-treated controls (~1.2-fold, *P*=0.2).

### Safety and tolerability of chronic Tf administration to mice

**Figure 3:**
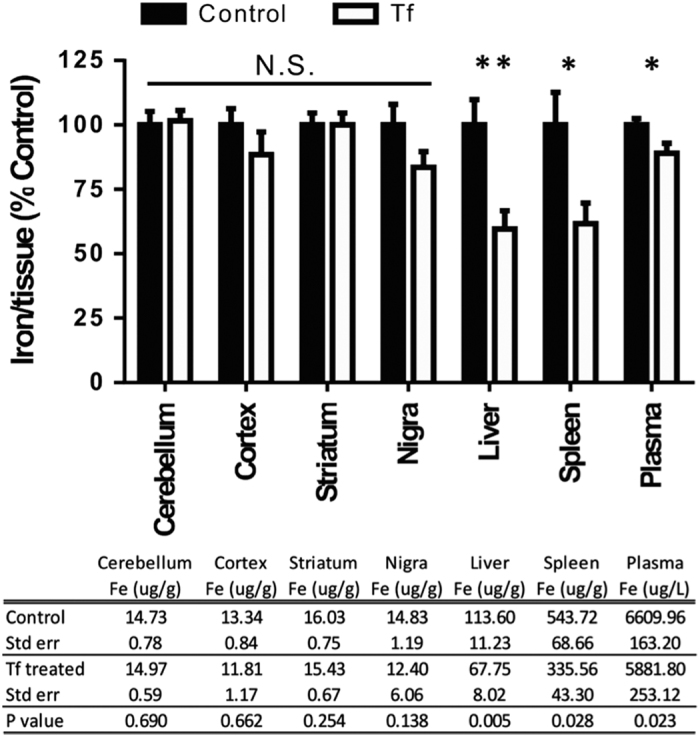
Chronic Tf supplementation causes iron depletion in peripheral tissues. C57/Bl6 mice were administered apo-Tf (*n*=9; 30 mg kg^−1^; subcutaneously) seven injections delivered every third day for 21 days and compared with controls (*n*=9). Perfused tissue was analyzed for iron by AAS. Data are means±s.e.m. *P<0.05, **P<0.01.

We investigated the effects of 21 day Tf treatment on systemic iron levels and other parameters in wild-type mice not administered MPTP. All mice survived treatment and there was no change in body weight (data not shown). After the treatment period, mice were killed and perfused, and the brain, liver, spleen and blood were removed and assayed for iron by AAS. There was no significant change in iron content in the brain regions analyzed after chronic apo-Tf treatment (Figure 3). In contrast to the brain, marked iron depletion was observed in the liver (−40%; *P*=0.005), spleen (−40%; *P*=0.028) and plasma (−12%; *P*=0.023) of chronic Tf-treated mice (Figure 3).

**Figure 4:**
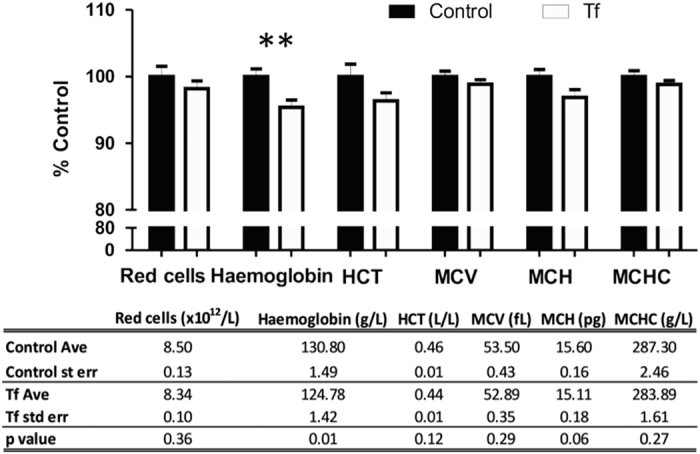
Chronic Tf supplementation decreases hemoglobin. C57/Bl6 male mice were administered apo-Tf (*n*=9; 30 mg kg^−1^; subcutaneously) every third day for 21 days and compared with controls (*n*=9). Blood was collected and hematology performed. HCT, hematocrit; MCV, mean corpuscular volume; MCH, mean corpuscular hemoglobin; MCHC, mean corpuscular hemoglobin concentration. Data are means±s.e.m. **P<0.01.

**Figure 5:**
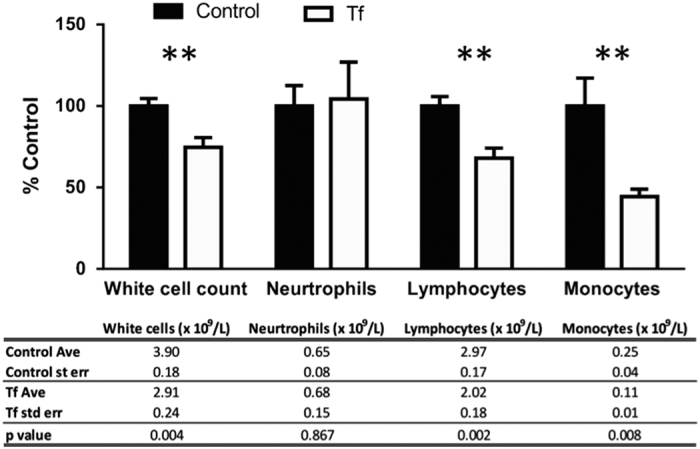
Chronic Tf supplementation causes depletion of white cells. C57/Bl6 mice were administered apo-Tf (*n*=9; 30 mg kg^−1^; subcutaneously) every third day for 21 days and compared with controls (*n*=9). Blood was collected and white cells were quantified by a pathology service. Data are means±s.e.m. **P<0.01.

As the iron depletion is often manifested as anemia, we examined the hematology profile of Tf supplemented mice. Indeed, Tf treatment lowered hemoglobin levels in blood (−5%; *P*=0.01, Figure 4). Other hematological parameters commonly affected in iron-deficient conditions (red cell count, hematocrit, mean corpuscular volume, mean corpuscular hemoglobin and mean corpuscular hemoglobin concentration), trended towards a decrease (Figure 3). We also investigated white cells in control and treated mice, as reduced lymphocyte proliferation is associated with iron chelation therapy.^5^ The total white cell count was decreased in Tf-treated mice (−25%; *P*=0.004; Figure 5), with a reduction in lymphocytes (−30%; Student’s two-tailed *t*-test: *P*=0.002) and monocytes (−55%; *P*=0.008; Figure 5).

## Discussion

Tf is the iron-transporting protein, with a high affinity to iron (10^−20^ M),^21^ able to deliver and remove iron from cells. The ability for Tf to act as the natural ligand for ferroportin-mediated iron export, while also being able to deliver iron through the TfR mechanism, made this protein an attractive candidate for a PD therapeutic where iron is elevated in a discrete nucleus (SN pars compacta^22^). We found that Tf was depleted in PD SN (Figure 1), which supports the possible use of Tf as a therapeutic for PD, and implicates Tf in the mechanism of disease. Tf was effective in removing iron from neuronal cells (Figure 2a), and in the MPTP model (Figures 2b–d); however, Tf supplementation depleted iron in healthy peripheral tissue (Figures 3, 4–5), thus not demonstrating advantage over traditional small-molecule chelators that are also complicated by off-target iron depletion.

The loss of Tf in PD SN (Figure 1) might contribute to iron accumulation in the disease. Reduced Tf has previously been reported in PD serum,^17^ where it has increased sialyation.^23^ Tf haplotype variability modifies the risk for PD.^24,25^ Further, Tf is mis-compartmentalized in the mitochondria of SN neurons in PD that could contribute to decreased Tf and the brain.^26^ The loss of Tf could restrict normal iron flux in the brain, as Tf is the major transporter of iron between cells. The loss of efficient inter-cellular transport could possibly explain why there is iron retention in some areas (SN^27^) and iron loss in other areas (temporal cortex^28^). In PD nigra, tau-mediated iron export is also compromised,^11,29,30^ and ceruloplasmin loading of Tf is disabled,^8,31^ which, together with the data presented here, collectively describe fatigue of iron export in the disease. Both ceruloplasmin and Tf supplementation were effective in the MPTP model, but Tf was less tissue selective in correcting iron overload.

Previous research has investigated Tf as a potential therapeutic agent for different diseases. Tf supplementation protects against iron toxicity in glial cells,^32^ and reverses some age-related declines in the weight of testis, thymus and adrenal gland.^33^ Tf supplementation has been shown to increase myelin deposition^34^ and increase the expression of myelin proteins^35^ that may be of benefit to multiple sclerosis, which is complicated by myelin deterioration with iron accumulation.^36^ Tf has also been explored in the treatment of various cancers.^37–43^ A cautious approach for using Tf as a therapeutic is advised in light of the results presented in the current study. These data support the exploration of agents that could correct the tau–ceruloplasmin–Tf iron export pathway in PD, but supplemental Tf may not be as selective as ceruloplasmin for use as a therapy. Alternatively, Tf therapy might prove more useful for other diseases of peripheral iron dyshomeostasis (for example, hemochromatosis).

Although we found that Tf, because of its off-target effects, was not an ideal therapeutic for PD, our data provide further support of iron chelation as a therapeutic strategy for PD. We show iron elevation in PD nigra (Figure 1), and the lowering of iron in cell culture and mice intoxicated with MPP+/MPTP was beneficial (Figure 2). These data support many pre-clinical studies that have shown the benefit of multiple iron-lowering drugs for PD (reviewed in Ayton *et al.*,^1^ and also a phase II clinical trial of PD.^2^ Iron chelation has been shown to benefit other neurological diseases in clinical trials, including Friedreich’s Ataxia,^44^ Neurodegeneration With Brain Iron Accumulation (NBIA)^45^ and Alzheimer’s disease.^46^ Iron therefore remains a promising therapeutic target for neurological diseases.
